# Physicochemical Properties, Stability, and Functionality of Non-Covalent Ternary Complexes Fabricated with Pea Protein, Hyaluronic Acid and Chlorogenic Acid

**DOI:** 10.3390/foods13132054

**Published:** 2024-06-27

**Authors:** Wenfei Fu, Fujun Liu, Ronglei Zhang, Ru Zhao, Yuxin He, Cuina Wang

**Affiliations:** Department of Food Science, College of Food Science and Engineering, Jilin University, Changchun 130062, China; fuwf9921@mails.jlu.edu.cn (W.F.); liufj9922@mails.jlu.edu.cn (F.L.); zhangrl9921@mails.jlu.edu.cn (R.Z.); zhaoru21@mails.jlu.edu.cn (R.Z.); yxhe23@mails.jlu.edu.cn (Y.H.)

**Keywords:** pea protein, hyaluronic acid, chlorogenic acid, non-covalent interactions, ternary complexes

## Abstract

The aim of this study was to prepare and characterize stable non−covalent ternary complexes based on pea protein (PP, 0.5%), hyaluronic acid (HA, 0.125%), and chlorogenic acid (CA, 0~0.03%). The ternary complexes were comprehensively evaluated for physicochemical attributes, stability, emulsifying capacities, antioxidant properties, and antimicrobial efficacy. PP-HA binary complexes were first prepared at pH 7, and then CA was bound to the binary complexes, as verified by fluorescence quenching. Molecular docking elucidated that PP interacted with HA and CA through hydrogen bonding, hydrophobic and electrostatic interactions. The particle size of ternary complexes initially decreased, then increased with CA concentration, peaking at 0.025%. Ternary complexes demonstrated good stability against UV light and thermal treatment. Emulsifying activity of complexes initially decreased and then increased, with a turning point of 0.025%, while emulsion stability continued to increase. Complexes exhibited potent scavenging ability against free radicals and iron ions, intensifying with higher CA concentrations. Ternary complexes effectively inhibited *Staphylococcus aureus* and *Escherichia coli*, with inhibition up to 0.025%, then decreasing with CA concentration. Our study indicated that the prepared ternary complexes at pH 7 were stable and possessed good functionality, including emulsifying properties, antioxidant activity, and antibacterial properties under certain concentrations of CA. These findings may provide valuable insights for the targeted design and application of protein-polysaccharide-polyphenol complexes in beverages and dairy products.

## 1. Introduction

Protein, polysaccharide and polyphenol participate in various non−covalent interactions, resulting in the formation of complexes critical for sensory attributes and functional characteristics of food products. Currently, there is a growing emphasis on elucidating the structural and functional properties of fabricated ternary complexes, driven by the overarching goal of enhancing food quality. Prepared ternary complexes can be used to avoid the degradation of polyphenols in adverse conditions, thereby exhibiting a beneficial protective effect [1]. Also, they have been shown to serve as functional materials that play a vital role in stabilizing emulsions, foams, and colloidal systems [2].

Pea protein (PP), as a promising alternative to conventional protein sources like animal protein and soy protein, has attracted great attention among researchers and industry. PP holds favorable attributes, including low allergenicity, non-genetically modified status, high nutritional value, widespread availability, and sustainability derived from crop cultivation. PP predominantly comprises 11S legumin, 7S vicilin and albumins [3]. PP contains high levels of lysine, which can help balance its deficiency in cereal−based diets [3]. PP exhibits various functional properties, including water and oil holding capacity, as well as emulsification, foaming, and gelling attributes [4].

Hyaluronic acid (HA), a linear macromolecular polysaccharide composed of alternating units of D-glucuronic acid and N-acetyl-D-glucosamine, boasts excellent hydrating, lubricating, viscoelastic, biodegradable, and biocompatible properties [5], making it one of the most versatile moisturizers and biomedical materials with extensive applications in pharmaceuticals, cosmetics, nutritional supplements, and as a novel food resource. Recently, extensive research has highlighted the potential of proteins and HA to form biopolymer complexes with distinctive functional properties under controlled binding conditions, offering numerous prospective applications. HA can form stable complexes with lactoferrin [6], whey protein [7], and bovine serum albumin [8], exhibiting superior physicochemical properties and facilitating the delivery of active substances.

Chlorogenic acid (CA), which comprises caffeic acid and quinic acid, is a common phenolic acid in foods such as honeysuckle, coffee beans, and citrus fruits. CA exhibits diverse biological activities, such as antioxidative and antimicrobial properties [9]. In recent years, CA has been used to formulate functional materials with protein and/or polysaccharide to improve the functional properties of macromolecules or endow complexes with good activity. A non-covalent ternary complex composed of whey protein concentrate, high-methoxyl pectin, and CA was synthesized in a previous study [10]. The complex efficiently protected CA against adverse environment. The complex demonstrated exceptional stability and superior antibacterial/antioxidant performance, suggesting its potential as an innovative product for commercialization.

This study aims to prepare and characterize stable non-covalent ternary complexes based on pea protein (PP, 0.5%), hyaluronic acid (HA, 0.125%), and chlorogenic acid (CA, 0~0.03%). Accordingly, the physicochemical attributes, stability, emulsifying capacities, antioxidant properties, and antimicrobial efficacy of these ternary systems were comprehensively evaluated.

## 2. Materials and Methods

### 2.1. Materials

Pea protein (PP, >85% protein based on a dry basis) was acquired from Shuangta Bio-tech Co., Ltd. (Shanghai, China). According to the manufacturer, the remaining ingredients in the PP powder were approximately 4.8% ash, 8.2% lipid, and 1.9% carbohydrate. Chlorogenic acid (CA, HPLC ≥ 98%) was obtained from Yuanye Biotechnology Co., Ltd. (Shanghai, China). Hyaluronic acid sodium (HA, purity of 95%, 20~60 kDa) was purchased from Bloomage Biotechnology Co., Ltd. (Shandong, China). Soybean oil was purchased from a local market (Changchun, China). 2,2-Diphenyl-1-picrylhydrazyl (DPPH) and 2,2′-azinobis (3-ethylbenzothiazoline-6-sulfonate) (ABTS) were purchased from Sigma-Aldrich (St. Louis, MO, USA). All other chemicals were commercially available and of analytical grade. The ultrapure water was obtained using Milli-Q equipment (Millipore, MA, USA).

### 2.2. Preparation of Soluble Protein

A soluble PP solution was prepared, utilizing methodologies established in a prior study [11], with certain modifications. PP powder was dissolved in ultrapure water and stirred for a duration of 2 h, following which the pH was adjusted to 12 and maintained under stirring for an additional 3 h. Subsequently, the pH was readjusted to 7. The PP solution was hydrated at 4 °C overnight and then centrifuged at respective speeds of 2000~7000 g for 20 min to remove insoluble aggregates. The accurate protein concentration was determined using a bicinchoninic acid (BCA) Kit. PP samples were determined for particle size according to Section 2.8.

### 2.3. Preparation of PP-HA Complexes

A PP solution was prepared, referring to the protocol outlined for the preparation of soluble proteins under centrifugation conditions of 6000× *g*. HA stock solution (0.5%, *w*/*v*) was formulated by dissolving it in ultrapure water, agitating for 2 h, and subsequently stored at 4 °C overnight to ensure complete hydration. PP, HA and PP−HA (mass ratio of 4:1) solutions at a concentration of 0.1% were adjusted from pH 8.5 to pH 1.5 at 0.5 intervals with NaOH or HCl.

### 2.4. Turbidity and Zeta-Potential

The turbidity of the samples prepared in Section 2.3 was assessed by measuring absorbance at 600 nm using a UV−visible spectrophotometer (UV2550, Shimadzu, Tokyo, Japan). The zeta-potential of each sample was determined using a Malvern Nano Zetasizer (Malvern Instruments Ltd., Malvern, UK) by measuring the direction and velocity of droplet movement in a U-shaped cuvette.

### 2.5. Preparation of Ternary Complexes

At pH 7, PP-HA binary non−covalent complexes (mass ratio of 4:1) with a protein concentration of 0.5% were prepared. CA powder was introduced into the binary complex solution, followed by readjustment to pH 7, and subsequently mixed at 700 rpm for 1 h to formulate the PP-HA-CA ternary non-covalent complex. Final concentrations of CA in the ternary system were 0.01%, 0.015%, 0.02%, 0.025%, and 0.03% (*w*/*v*).

### 2.6. Fluorescence Spectroscopy

The CA stock solution (4 mM) was added to PP-HA complex solutions (pH 7, 0.1%) gradually to achieve various CA concentrations (0, 10, 20, 30, 40, 50, and 60 μM), followed by adjusting the solution to pH 7. The complexes were kept for 1 h on a magnetic stirrer (700 rpm) in the absence of light until further analysis. Intrinsic fluorescence spectra of samples were measured using a RF-5301 Fluorometer (Shimadzu, Tokyo, Japan) at an excitation wavelength of 280 nm and an emission wavelength scope of 290~500 nm. The binding mechanism, binding constant (K_a_), and binding stoichiometry (n) of the complexes were determined utilizing Stern−Volmer and double logarithmic equations, according to our previous study [12].

The expressions for the Stern-Volmer and double logarithmic equations are as follows.

(1) $$\frac{F_{0}}{F}=1+K_{q}\times \tau_{0}\times \left[\mathrm{CA}\right]=1+K_{\mathrm{sv}}\times \left[\mathrm{CA}\right]$$

(2) $$\log\left[\frac{\left(F_{0}-F\right)}{F}\right]={\mathrm{logK}}_{a}+\mathrm{nlog}\left[\mathrm{CA}\right]$$

where F_0_ and F are the fluorescence intensities of PP samples without and with CA, respectively. K_q_ is the bimolecular quenching constant and τ_0_ is the average lifetime of PP without any quencher that is 10^−9^ s. K_a_ is the binding constant and n is the binding number.

### 2.7. Molecular Docking

11S Legumin (3KSC) and 7S vicilin (6L4C), as the primary constituents of PP, were selected as model proteins, and their structures were downloaded from the Protein Data Bank (https://www.rcsb.org (accessed on 8 May 2024)). Molecular structure files of HA monomer (CID: 6852395) and CA (CID: 1794427) were obtained from PubChem (https://pubchem.ncbi.nlm.nih.gov (accessed on 8 May 2024)). Subsequently, both small molecules and proteins were preprocessed using PyMOL (Version 2.4.0.) software to eliminate ligands and water molecules, and hydrogen atoms were added. Ligand-receptor docking was conducted employing AutoDock Tools-1.5.7, following a methodology outlined in prior research [13]. Initially, Gasteiger charges were assigned to the proteins, subsequent to which AutoGrid was executed to compute the energy within the grid points. Finally, AutoDock was employed to execute the conformational search and evaluation. The binding conformations of ligands and the surrounding amino acids at their respective binding sites were visualized using PyMOL software.

### 2.8. Particle Size and Zeta-Potential

Samples were determined for particle size and zeta−potential using a Malvern Nano Zetasizer. The sample was determined for particle size and Polydispersity Index (PDI) at a backscatter detection angle of 173°. The zeta-potential of each sample was determined were determined according to Section 2.4.

### 2.9. Stability Measurement

Newly prepared complexes were utilized to investigate the thermal stability and photostability. All ternary complex solutions underwent heating at 85 °C for 30 min or were subjected to UV light (365 nm) exposure at room temperature for 2, 4, 8, 12, and 24 h. The stability of samples was assessed with parameters of particle size, zeta-potential, and CA retention.

### 2.10. CA Retention Measurement

CA was extracted from ternary complex suspension through a 20−min ultrasonication process, followed by centrifugation at 14,000× *g* for 20 min to precipitate PP and HA. Resultant supernatant containing CA was diluted tenfold, and its absorbance was measured at 324 nm. The concentration of CA in the supernatant was accurately determined, utilizing the previously constructed CA standard curve (R^2^ > 0.99). The retention rate is defined as the ratio of the concentration of CA after processing to the initial concentration of CA.

### 2.11. Emulsifying Properties

Following the methodology outlined in a previous study [14], with minor adjustments, soybean oil (W/O = 9:1, *v*/*v*) was introduced into each ternary complex aqueous solution. Crude emulsions were generated by subjecting samples to emulsification using a high−speed shear mixer (T25 digital ULTRA-TURRAX, IKA, Staufen, Germany) operating at 12,000 rpm for 2 min. At intervals of 0 and 10 min, 50 μL solution was extracted from the bottom of centrifuge tube. Subsequently, emulsions were diluted with a 0.1% (*w*/*v*) SDS solution, and absorbance was measured at a wavelength of 500 nm, denoted as A_0_ and A_10_, respectively. The emulsifying activity index (EAI) and emulsion stability index (ESI) were calculated according to the previous study [14]. Simultaneously, particle size and zeta−potential of the emulsion were assessed. The EAI and ESI were calculated as follows.

(3) $$\mathrm{EAI}\;\left(m^{2}/g\right)=2\times 2.303\frac{A_{0}\times N}{C\times \varphi \times 10,000}$$

(4) $$\mathrm{ESI}\;\left(\%\right)=\frac{A_{10}}{A_{0}}\times 100\%$$

where N represents the dilution factor, C represents the sample concentration (g/mL), φ represents the volume fraction of the oil phase in the composite emulsion, and A_0_ and A_10_ represent the absorbance values at 0 min and 10 min.

### 2.12. Antioxidant Activity

#### 2.12.1. DPPH/ABTS Scavenging Capacity

Samples underwent assessment for their antioxidant properties with certain modifications utilizing the methodology outlined in our previous study [15]. A 2,2-Diphenyl-1-picrylhydrazyl (DPPH) solution (0.2 mM) was prepared by dispersing the powder into methanol. Diluted samples were mixed with the DPPH solution at a volume ratio of 3:1 and incubated in the dark for 30 min. The absorbance of all solutions was then measured at 517 nm using a microplate reader (Synergy HT, BioTek, Winooski, VT, USA).

A 2,2′-Azinobis (2-ethylbenzothiazoline-6-sulfonate) (ABTS) radical solution was prepared by mixing ABTS (7 mM) and potassium persulfate (2.45 mM) in a 1:1 volume ratio and stored in the dark for 12 h. Diluted samples were blended with 150 μL ABTS radical and incubated for 6 min. Absorbance was measured at 734 nm.

According to the following equation, the scavenging activity of DPPH and ABTS∙ was calculated.

(5) $$\mathrm{SA}\;\left(\%\right)=1-\frac{A_{S}-A_{B}}{A_{R}}\times 100\%$$

where A_S_, A_B_, and A_R_ represent the absorbance of sample with DPPH/ABTS∙, the blank, and the DPPH/ABTS∙ solution, respectively.

#### 2.12.2. Ferric Reducing Ability Power (FRAP)

The iron-reducing capacity of complexes was assessed employing a methodology based on the protocol delineated in a previous study [16], with minor adaptations. Samples were combined with a potassium ferricyanide solution (0.5%, *w*/*v*) at a 1:5 ratio, followed by incubation at 50 °C for 20 min. Subsequently, trichloroacetic acid (10%, *w*/*v*) was added to remove interference. After centrifugation at 6000× *g* for 10 min, the diluted supernatant was mixed with a ferrous chloride solution (0.1%, *w*/*v*). Following further incubation, the absorbance was measured at 700 nm.

### 2.13. Antimicrobial Activity

The antimicrobial activity of the samples against *Staphylococcus aureus* and *Escherichia coli* was evaluated. Initially, 100 μL of nutrient broth solution containing bacteria and 100 μL of samples diluted fivefold (0~60 mg/L) were inoculated into a 96-well plate to ensure an initial bacterial concentration of 1 × 10^6^ CFU/mL. Subsequently, the plate was incubated at 37 °C for 12 h, and the absorbance at 600 nm of the samples was measured hourly using a microplate reader.

### 2.14. Statistical Analysis

All experimental results, except for fluorescence curves and molecular docking results, were reported as mean ± SD, obtained from the average of triplicates for at least two batches. Statistical analysis was performed using SPSS software (SPSS Inc. Chicago, IL, USA). The significant differences (*p* < 0.05) reported in the experimental results were determined using one-way analysis of variance followed by the Least Significant Difference (LSD) method.

## 3. Results and Discussion

### 3.1. Selection of the Preparation Conditions for Soluable Pea Protein Solution

**Figure 1:**
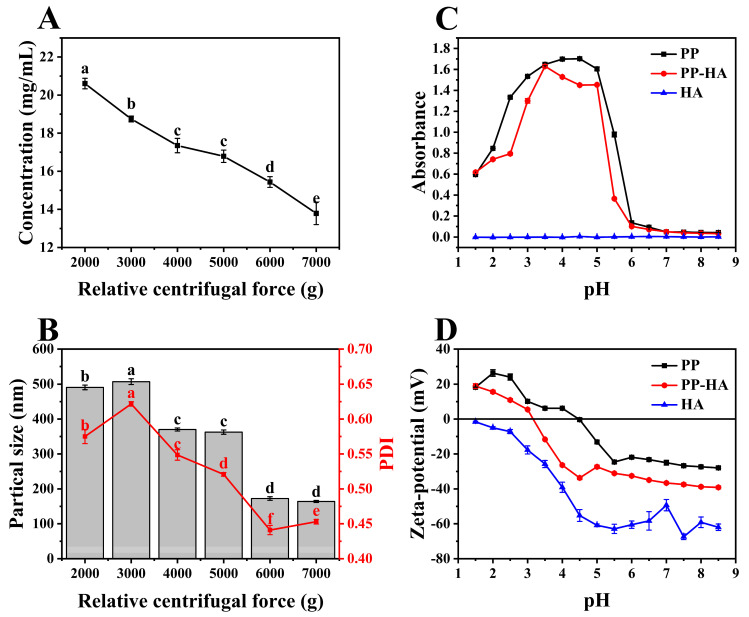
Protein concentration (**A**), particle size, and polydispersity index (PDI) (**B**) of pea protein supernatant under different centrifugation conditions. Absorbance at 600 nm (**C**) and zeta−potential (**D**) of PP, HA and PP−HA mixture during acidification. Concentration of PP, HA and PP−HA mixture was 0.1% (*w*/*v*). Different letters indicate a significant difference (*p* < 0.05).

Freshly prepared PP solution often contains large aggregates, which will generate precipitation quickly. Therefore, centrifugation was often utilized to remove the insoluble particles. Based on the varying centrifugation forces utilized in prior studies, the range of centrifugation conditions (2000~7000 g) has been established [11,17]. To select a treatment conditions, the effects of centrifugation conditions on protein concentration and particle size, along with PDI in PP supernatant were studied and results are shown in Figure 1A,B. The protein concentration in the PP supernatant before centrifugation was approximately 25.28 mg/mL, suggesting that the solubility of PP after pH-shifting treatment was about 63% (the original concentration was 4%). The pH-shifting dispersion of insoluble aggregates leads to the breaking of some non-covalent bonds and thus increases the solubility [18]. As centrifugation force increased, the concentration significantly decreased from 20.61 mg/mL to 13.79 mg/mL (*p* < 0.05), which suggested that more protein particles separated and precipitated from the liquid phase. Additionally, at higher centrifugal forces, protein particles were more prone to aggregation, which was more easily precipitated.

Generally, particle size and PDI of samples decreased with increasing relative centrifugal force, and the lowest values were observed at 7000 g and 6000 g, respectively. However, no significant difference in particle size was observed between centrifugal forces of 6000 g and 7000 g. The dissolved proteins in the supernatant after pH-shifting were mainly albumin with a small monocular weight, and some insoluble globulins (convicilin, vicilin, and legumin) were converted into the soluble proteins [19]. Centrifugal force separated solid protein particles from the system, and conformation may be influenced by the centrifugation process. This also suggested that protein solutions with small diameters exhibited heightened stability, potentially exposing a greater number of binding sites on their surface. Considering the changes in protein concentration, particle size, and PDI, alongside economic and environmental factors, 6000 g was chosen to prepare soluble PP solutions for further analysis, which had a protein concentration of 15.43 mg/mL, particle size of 172 nm and PDI of 0.44.

### 3.2. Preparation of PP-HA Mixture

Turbidity and zeta-potential serve as indicators of electrostatic complexation between protein and polysaccharide during acidification. Change in turbidity of PP, HA, and PP-HA throughout the acidification procedure from pH of 8.5 to 1.5 is elucidated in Figure 1C. The turbidity of PP initially rose then fell, peaking at near pH 4.5 due to its proximity to the isoelectric point, causing self-aggregation and precipitation, in consonance with antecedent scholarly investigations [20]. The optical property of HA was negligible due to its strong intermolecular repulsion, which cannot scatter light strongly [21]. PP-HA mixture showed overall lower turbidity below PP with peak at around pH 3.5. This observation suggests that the presence of HA diminished and delayed the aggregation of PP through electrostatic interactions, thereby conferring stability to the system.

Critical pH values associated with the structural transition of PP-HA can be estimated through turbidimetric analysis [13]. Turbidity increase is generally due to component complexation, while decrease signifies complex dissociation [22]. Observing the trend of turbidity curves, it was found that soluble complexes formed around pH 7 due to the electrostatic interaction between PP and HA. At around pH 6, insoluble complexes began to form due to strong electrostatic attraction. The turbidity peaked at pH 3.5, indicating the maximum interaction strength. As the pH further decreased, turbidity started to decrease due to the disintegration of large complexes.

Surface charge measurements offer insights into biomolecular electrostatic interactions, indicating mixed dispersion system stability. Medium pH can alter the surface charge properties of proteins and polysaccharides, impacting their interaction and complex formation or dissolution [23]. Figure 1D elucidates the evolution in surface charge of PP, HA, and PP-HA during the acidification process. PP showed zeta-potential from −27.95 mV at pH 8.5 to 18.23 mV at pH 1.5 due to protonation of -NH^3+^ and deprotonation of -COO^−^. The zeta-potential of PP became neutral (0 mV) at about pH 4.5, aligning with its documented isoelectric point [24]. HA displayed an initial zeta-potential of −62.00 mV (pH 8.5) and maintained the negative charge across the whole tested pH range due to the -COO^−^ distribution on the main chain. The charge curve of PP-HA lay between those of PP and HA, indicating the presence of electrostatic interactions between the two polymers. The introduction of HA contributed to an increase in the negative charge within the system. At a pH of approximately 3.5, the potential became neutral, corresponding to the maximum turbidity. When the pH is below the isoelectric point of PP and exceeds the pK_a_ of HA, opposite surface charges occur, fostering electrostatic interactions [25]. Even beyond pH 4.5, PP can still bind with HA due to the charge patch model, leveraging the protein’s uneven charge distribution [26].

### 3.3. Binding Ability of PP-HA to CA

Stable binary PP-HA complexes were prepared at pH 7, according to the established turbidity curve of the mixture as a function of pH value. Then CA at various concentrations was incorporated.

**Figure 2:**
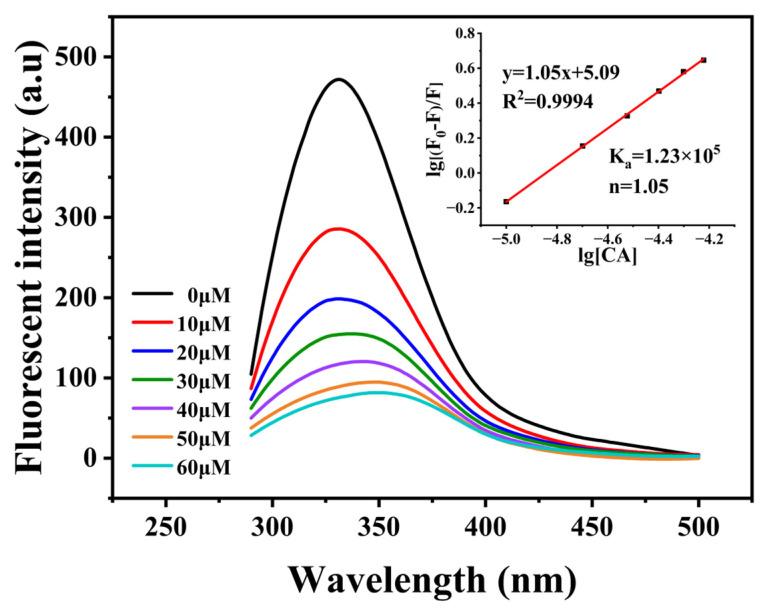
Fluorescence emission spectra of PP−HA complex (0.1%, *w*/*v*) with different concentrations of CA at pH 7. Inserted Figure indicates linear relationship between lg [(F_0_ − F)/F] and lg [CA]. F_0_ and F represent the maximum fluorescence intensity of PP−HA alone or in combination with CA.

Monitoring the fluorescence intensity and wavelength changes of aromatic amino acids at an excitation wavelength of 280 nm can elucidate structural alterations in proteins upon binding with polyphenols, while fluorescence quenching experiments can be employed to investigate the binding affinity between protein and small ligand. Figure 2 shows the fluorescence behavior of the PP-HA complex with a gradient concentration of CA under an excitation wavelength of 280 nm. The spectrum of PP−HA revealed a maximum emission peak at 330 nm. After the addition of CA, a red shift in the maximum emission peak was observed, indicating the formation of a ternary complex. Furthermore, this change also signified alterations in the tertiary structure of the protein, suggesting the exposure of tryptophan residues and protein unfolding, with fluorescent groups transitioning from a predominantly hydrophobic environment to a more hydrophilic one.

Simultaneously, a notable decrease in fluorescence intensity of PP−HA was observed upon the addition of CA. The reduction suggested the quenching of the aromatic fluorescence of PP induced by CA. Fluorescence quenching can result from various processes, including molecular collisions, ground−state complex formation, or energy transfer [27], which is commonly classified into dynamic and static quenching types. To investigate the quenching mechanism, intrinsic fluorescence data were analyzed using the Stern–Volmer equation. The obtained bimolecular quenching constant (K_q_) of 7.23 × 10^13^ L/mol/s significantly exceeded the maximum dynamic quenching constant (2.0 × 10^10^ L/mol/s), indicating a static quenching mechanism and the formation of a ternary complex [28].

Intrinsic fluorescence data were analyzed to determine the binding constant (K_a_) and number (n). K_a_ between PP−HA and CA was 1.23 × 10^5^ L/mol, with a binding number of 1.05. The interaction between PP and CA reported in the previous study [29] showed binding constants and numbers of 3.41 × 10^4^ L/mol and 0.93, respectively. In contrast, the interaction between HA and PP did not alter the types of binding sites for CA on PP but changed the binding strength. This suggests that the binding of CA to PP may be enhanced by HA, thus providing a theoretical basis for the development of stable ternary systems.

### 3.4. Molecular Docking

Molecular docking analysis, based on the identification of various ligand conformations within the active site of a protein and subsequent ranking of these conformations by binding affinity, furnishes valuable data concerning protein-ligand interactions [30]. Previous studies have demonstrated the feasibility of utilizing monomeric HA and CA molecules for molecular docking analysis with proteins, enabling the investigation of small molecule-protein binding interactions [31,32].

**Figure 3:**
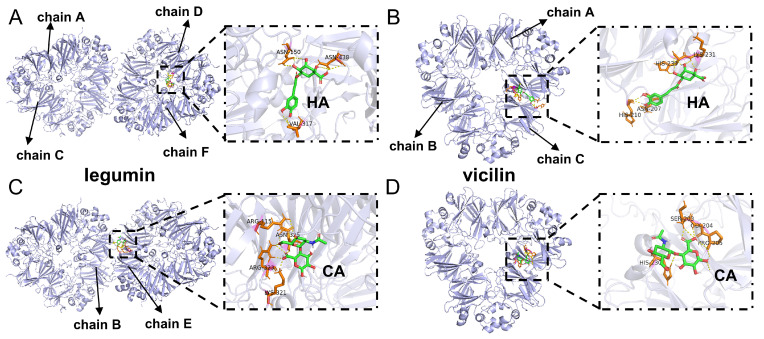
Legumin docking sites with HA (**A**) and CA (**C**), and vicilin docking sites with HA (**B**) and CA (**D**), along with amino acids involved in hydrogen bonding.

HA was docked into legumin and vicilin, and the results are depicted in Figure 3A,B. HA was docked at the interface of the E and B chains of legumin and at the midsection of the C chain of vicilin, respectively. The binding energies for legumin and vicilin with HA were −4.85 kcal/mol and −3.56 kcal/mol. This suggested that HA could bind to both legumin and vicilin, with a higher affinity for legumin. Upon further examination of the specific energy values from the docking results in Table 1, it was revealed that hydrogen bonding, hydrophobic interactions, and electrostatic interactions played a significant role in facilitating the binding of HA to PP. HA formed five and four hydrogen bonds with legumin and vicilin, respectively. In legumin, the interactions involved ASN325, ARG323, LYS321, and ARG115 of the E chain, whereas in vicilin, they involved LYS231, PRO205, SER203, and GLY204 of the C chain. Hydrogen bonds likely form between the hydroxyl and carboxyl groups on HA and the amino groups on the protein.

CA underwent docking with legumin and vicilin, and the outcomes are illustrated in Figure 3C,D. CA was docked at the interface of the C and D chains of legumin, and considering the docking results of HA, this indicated a high affinity at the peptide chain junction in legumin. The fact that HA and CA bind to different sites on legumin implies the potential formation of a ternary complex. Additionally, CA was similarly docked at the midsection of vicilin’s C chain, partially overlapping with HA’s binding site, indicating a region of high activity on vicilin. Simultaneously, HA and CA, at similar spatial positions, likely form a ternary complex through hydrogen bonds and hydrophobic interactions. The binding energies of legumin and vicilin with CA were −5.39 kcal/mol and −5.55 kcal/mol, respectively, indicating that CA could bind to both legumin and vicilin with similar binding affinities. The specific energy values from the docking results (Table 1) revealed that the interaction between CA and PP predominantly relied on hydrogen bonding and hydrophobic interactions, forming four hydrogen bonds each. In legumin, these interactions involved VAL317 and ASN150 of the F chain, along with ASN438 of the D chain. Simultaneously, in vicilin, hydrogen bonds were formed with HIS230, LYS231, HIS210, and SER203 of the C chain. On CA, an abundance of hydroxyl groups are poised to engage in hydrogen bonding with the amino and carboxyl groups present on the protein.

### 3.5. Particle Size and Zeta-Potential of Ternary Complexes

**Figure 4:**
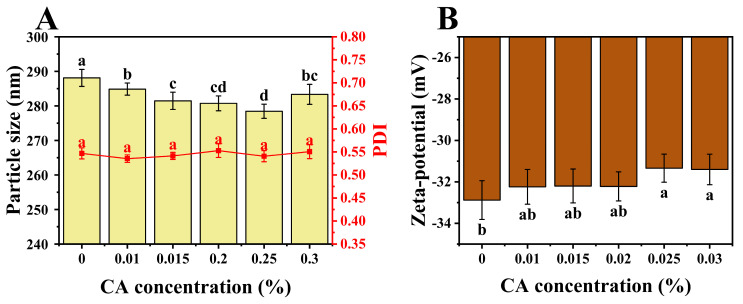
Particle size and polydispersity index (PDI) (**A**), and zeta−potential (**B**) of pure PP−HA and its complexes with different concentrations of CA. Different letters indicate a significant difference (*p* < 0.05).

Particle size and zeta-potential were measured for ternary complexes, and the results are shown in Figure 4A,B. All samples had particle sizes ranging from 278 nm to 288 nm, indicating the formation of nanoscale composites. Nanoparticles possess distinctive characteristics such as a large surface area, high reactivity, and strong permeability. They are easily prepared, exhibit high payload capacity, and demonstrate superior physicochemical stability [33]. Consequently, they hold considerable promise for extensive applications in the functional food and pharmaceutical industries. At pH 7, both PP and HA polymers carry negative charges, resulting in relatively weak interactions between them. Consequently, both PP and HA are likely to bind with CA, thereby influencing the particle size of the complexes [34]. The particle size initially decreased, reaching a minimum (278 nm) around 0.025% CA concentration (with no significant difference observed between 0.025% and 0.02% CA concentrations), followed by an increase. The reduction in particle size can be attributed to the expulsion of water from the complex, leading to particle shrinkage [35]. This effect may primarily result from the binding of PP with CA, causing a tightening of the PP structure with the embedding of hydrophobic sites, thus expelling water. Simultaneously, hydrophobic interactions between HA and CA could also lead to the loss of some water molecules. At a CA concentration of 0.025%, the potential binding sites on the protein may become saturated, leading to mutual repulsion between free and bound CA molecules. This diminishes CA’s interaction with PP, allowing for partial structural recovery and reassociation with water molecules, thereby increasing particle size.

Figure 4B demonstrates that all samples had absolute zeta−potential above 30 mV, which suggests a stable colloidal system [36]. With increasing CA concentration, the absolute zeta-potential of the complex exhibited a decreasing trend, with no significant differences (*p* < 0.05) observed between all concentrations. Since the overall change in potential is not significant, it can be inferred that electrostatic interaction may not be the primary driving force between PP-HA and CA. The decreased surface charge of ternary complexes in comparison with binary complexes is the combined result of the following factors. At pH 7, which is higher than the pK_a_ of CA, deprotonation of the phenolic groups of CA may occur, generating oxygen centers with high negative charge density, leading to a decrease in the zeta−potential of the samples [37]. However, the hydroxyl groups of CA can form hydrogen bonds with the carboxyl groups of proteins [38], which can reduce the negative surface charge on the protein. Based on the results, it was evident that the dominant effect led to a decrease in negativity.

### 3.6. Stability Analysis

#### 3.6.1. Thermal Stability

**Figure 5:**
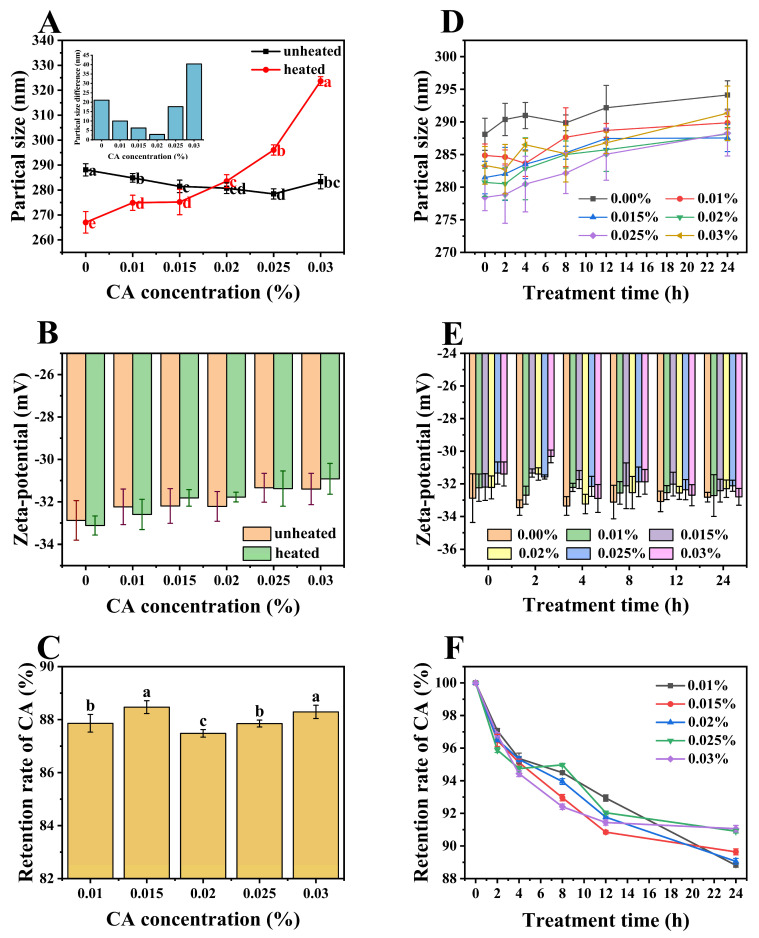
Before and after heat treatment at 85 °C for 30 min, and during UV irradiation for 24 h, the particle size (**A**,**D**) and zeta potential (**B**,**E**) of PP−HA and PP−HA−CA ternary complexes with different CA concentrations, along with the retention rate (%) of CA (**C**,**F**) in the ternary complexes. The inserted Figure of (**A**) indicates the difference in particle size before and after sample heat treatment. Different letters indicate a significant difference (*p* < 0.05).

Thermal treatment will disrupt the equilibrium of interactions among components, thus affecting the overall stability of the colloidal system. All ternary complexes were subjected to heat treatment at 85 °C for 30 min, following common sterilization protocols in the food industry. As shown in Figure 5A, the particle size of untreated samples initially decreased with increasing CA concentration before subsequently increasing, whereas the particle size of heat−treated samples exhibited a consistent increase throughout. Heat treatment induces conformational changes in proteins, revealing previously concealed hydrophobic sites and consequently augmenting the protein surface hydrophobicity [39]. This may facilitate hydrophobic interactions between proteins and nonpolar molecules [40], leading to an increase in particle size. Moreover, the heat treatment could also prompt the formation of covalent cross−links or complexes between proteins and CA, further influencing the particle size variation. Both effects caused the particle size to increase as the CA concentration increased. In comparison to untreated samples, PP-HA-CA complexes with CA concentrations below 0.02% exhibited smaller particle sizes, whereas those with CA concentrations equal to or above 0.02% showed larger particle sizes. Therefore, as CA concentration increased, the particle size change before and after heat treatment was initially large, then decreased, and then increased again, with the smallest change occurring around 0.02%, indicating the highest stability. The reason for this phenomenon is that, as a hydrophilic colloid molecule, HA might act as a blocking agent at specific temperatures [41], impeding protein aggregation. Therefore, at lower concentrations, the particle size of heat-treated samples is reduced compared to untreated samples. Between CA concentrations of 0.015% and close to 0.02%, a delicate equilibrium may be achieved at a specific threshold, wherein the opposing forces governing particle size augmentation and diminution are balanced, thereby resulting in a consistent particle size before and after heat treatment, exhibiting maximum stability.

Changes in the zeta-potential of samples before and after thermal treatment are illustrated in Figure 5B. The absolute potential of both heated and unheated samples decreased with the rise in CA concentration. Simultaneously, at the same CA level, there was no significant difference (*p* < 0.05) observed between heated and unheated samples, and all potential values ranged from −33.12 mV to −30.92 mV, indicating that thermal treatment had minimal influence on the surface charge of molecules and electrostatic interactions within the system, resulting in stable colloidal systems.

Due to the unsaturated bonds present in its molecular structure, CA is vulnerable to adverse environmental factors. The introduction of energy through heat treatment may disrupt intermolecular interactions and induce conformational changes in proteins, consequently leading to the release of a fraction of CA molecules, which may undergo degradation. As shown in Figure 5C, after heat treatment, the retention rates of CA in the samples were relatively high, ranging from 87% to 88%, indicating the strong thermal stability of CA in the ternary system. This phenomenon may be attributed to the protective effect of PP-HA on CA. Retention rates of CA among the samples exhibited little variation, with no apparent change pattern. The minimal variation in retention rates among the samples may stem from the pronounced thermal degradation effect on CA. As more CA was introduced, a proportional increase in degradation occured, resulting in comparable levels of ultimate degradation.

#### 3.6.2. UV Stability

When exposed to UV light, as depicted in Figure 5D, with longer exposure, the particle size of both binary and ternary complexes increased similarly, suggesting that PP−HA primarily determines the photostability of the system. Exposure to UV light induces conformational changes in proteins, resulting in structural relaxation or a partial loss of stability within the complexes [42]. This irradiation may also trigger oxidation reactions within the complexes, thereby altering their chemical and structural properties.

According to Figure 5E, the potential of the ternary system remained relatively stable under UV light exposure. This stability might have stemmed from protein denaturation, causing the exposure of hydrophobic groups and a reduction in surface hydrophilic groups, resulting in an increase in negative potential. Concurrently, the decomposition of CA into quinic acid and caffeic acid might have offset the negative impact on the potential [43]. Additionally, UV light might have partially decomposed CA, generating reactive species or free radicals that interacted with PP−HA complexes, inducing changes in protein conformation or chemical modifications, thereby partially affecting potential variation. Simultaneously, it was observed that under prolonged UV exposure, there was a decrease in the zeta−potential difference among samples with varying CA concentrations. This phenomenon suggested a diminishing influence of CA, potentially attributable to the UV−induced partial decomposition of CA.

Based on the experimental data, it was observed that after 24 h of UV light exposure, the maximum particle size variation among different concentrations of CA complexes was 9.82 nm. The range of potential values was 3.14 mV, all of which were below −30 mV. These findings indicate the excellent physical stability of the ternary system under UV light exposure.

Retention rates of CA in PP-HA-CA complexes under UV light irradiation were measured (Figure 5F). CA in all samples exhibited varying degrees of degradation, and the retention rates remained above 88% after 24 h, indicating high photostability of CA within the complexes. Furthermore, it was observed that in samples with higher CA concentration, the variation in CA retention rates was relatively smaller. This phenomenon could potentially be attributed to a limited amount of CA being degraded per unit time [44], resulting in a non-proportional degradation of the added amount.

### 3.7. Emulsifying Properties

**Figure 6:**
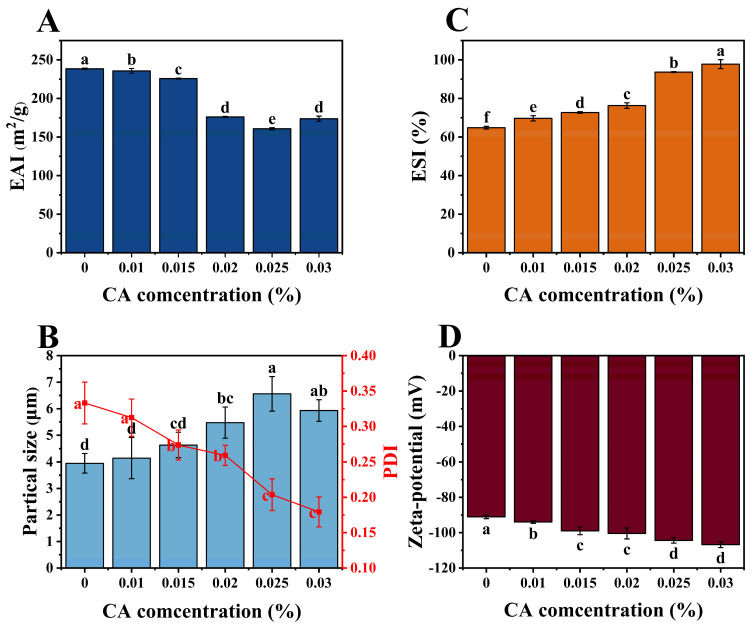
EAI (**A**) and ESI (**C**) of pure PP−HA and its complexes with different concentrations of CA, along with the particle size and PDI (**B**), and zeta-potential (**D**) of the crude emulsions formed. EAI is emulsifying activity index and ESI is emulsion stability index. Different letters indicate a significant difference (*p* < 0.05).

EAI and ESI are common indicators of protein−based emulsifiers’ properties. EAI reflects protein adsorption at the interface, while ESI indicates its ability to stabilize emulsions [45]. As shown in Figure 6A, EAI initially decreased then rose with increasing CA concentration, reaching a minimum at 0.025%. This trend is inversely correlated with emulsion droplet size variation, as depicted in Figure 6B. Combining the previous speculations, the trends in EAI and emulsion droplet size variation can be explained as follows: Upon addition of CA, it interacted with the hydrophobic sites of the protein, resulting in a denser complex where hydrophobic groups were embedded. Additionally, CA introduced polar groups such as hydroxyl and carboxyl, thereby increasing the hydrophilicity of the complexes [46], and altering the hydrophilic-lipophilic balance (HLB value) of the complexes to some extent. With increasing CA concentration, this effect became more pronounced. When the CA concentration reached 0.025%, saturation was achieved in the binding of PP with CA, and electrostatic interactions led to partial restoration of the protein’s conformation and surface hydrophobicity. This process is closely associated with EAI and is crucial for the emulsification of oil and water. The correlation between EAI trends and the particle size of the ternary complex confirm the influence of CA concentration on complex behavior. Consequently, the particle size of emulsion droplets was inversely proportional to the hydrophobicity of the complex, which correlated negatively with EAI [47].

As depicted in Figure 6C, ESI increased with the rising concentration of CA, while Figure 6D showed a corresponding increase in the emulsion’s negative zeta-potential. This highlighted an obvious positive correlation between absolute potential and ESI. ESI was facilitated by the increase in absolute potential, which strengthened electrostatic repulsion among particles, ensuring dispersion and inhibiting aggregation. Upon the addition of CA, the emulsion’s absolute potential increased due to several interrelated mechanisms. Initially, CA may attach to certain positively charged amino acid residues or induce intermolecular/intramolecular cross−linking, leading to the masking of positively charged residues and consequently resulting in an increase in the abundance of negative charges [48]. Moreover, the inherent carboxyl or hydroxyl groups in CA may induce an increase in the electrostatic charges of PP molecules, thereby enhancing both intra- and intermolecular electrostatic repulsion and resulting in an increase in absolute potential [49]. Furthermore, CA may induce alterations in the interfacial properties between the aqueous and oil phases, resulting in the redistribution of surface charges and subsequent modulation of the emulsion’s zeta−potential. Additionally, as illustrated in Figure 6B, the PDI of the emulsion droplets decreased with increasing CA concentrations. This indicates a more uniform distribution of droplet sizes and reflects enhanced stability of the emulsion system.

### 3.8. Antioxidative Properties

PP possesses distinct antioxidant properties via hydrogen or electron transfer mechanisms due to containing the amino acids His, Phe, and Gln [50]. HA also exhibits antioxidant properties with hydroxyl groups. CA scavenges free radicals and hinders chain reactions by forming stable semiquinone radicals. Moreover, it acts as an antioxidant by reducing Fe^3+^ and scavenging both chemical and reactive oxygen species [51].

**Figure 7:**
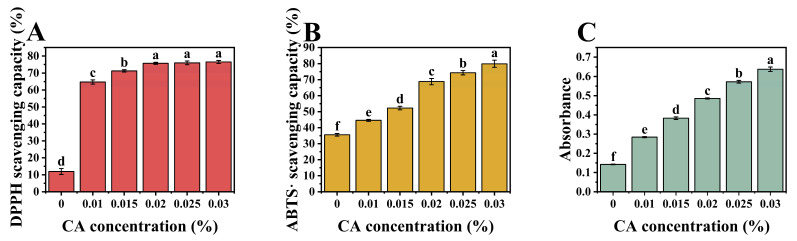
Antioxidant activity of pure PP-HA and its complexes with different concentrations of CA, including DPPH radical scavenging activity (**A**), ABTS∙ radical scavenging activity (**B**), and Ferric reducing ability power (measured as absorbance at 700 nm) (**C**). Different letters indicate a significant difference (*p* < 0.05).

As the concentration of CA increased, as depicted in Figure 7A, there was a significant rise in DPPH radical scavenging activity (*p* < 0.05), which saturated at 0.02%. Concurrently, Figure 7B exhibited a linear increase in ABTS· scavenging activity. Additionally, Figure 7C indicated increased absorbance values, signifying enhanced iron reduction capacity. The inherent capacity of CA to scavenge free radicals and inhibit oxidation, as well as its potential to form stable antioxidant complexes by interacting with amino acid residues or HA molecules within PP−HA, both contribute to the enhancement of the overall antioxidant activity of the system with increasing CA concentration. When considering the saturation of DPPH scavenging activity at 0.02%, two interrelated conjectures arose. Firstly, the DPPH solution was dissolved in methanol, and during the resting period, PP might have undergone dehydration-induced denaturation, leading to premature saturation of binding sites. Additionally, free CA molecules might have undergone excessive solvation by polar solvent molecules, hindering their antioxidative efficacy. Secondly, according to previous research [52], the mechanisms of antioxidative action are categorized into hydrogen atom transfer (HAT) and single electron transfer (SET). While both DPPH and ABTS· assays operate via a mixed mode (HAT and SET), DPPH tends towards the HAT mechanism, whereas the FRAP assay is based on SET. It is conjectured that the antioxidative activity of the system results from a combined action of HAT and SET mechanisms. At a CA concentration of 0.02%, saturation of CA binding sites on PP leads to particle shrinkage and a tight complex structure, rendering hydrogen atom transfer difficult and thus resulting in saturation of DPPH scavenging activity.

### 3.9. Antimicrobial Activity Analysis

**Figure 8:**
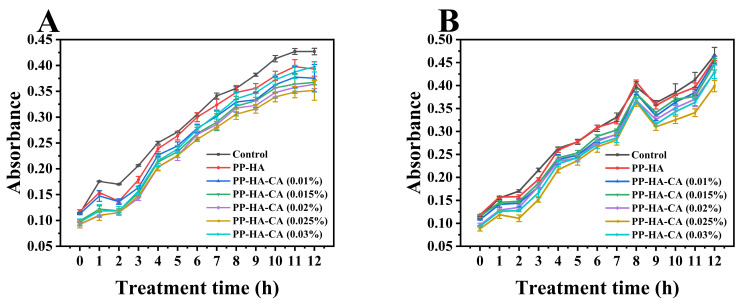
Growth curve (measured as optical density at 600 nm) of *Staphylococcus aureus* (**A**) and *Escherichia coli* (**B**) in the presence of PP-HA complexes and PP−HA complexes with different concentrations of CA.

*Staphylococcus aureus* and *Escherichia coli*, representing Gram-positive and Gram−negative bacteria, are common foodborne pathogens. Changes in the optical density of nutrient broth solutions containing bacteria were measured to assess bacterial growth for *S. aureus* and *E. coli* under sample-treated and blank control conditions, and the growth curves are depicted in Figure 8. In comparison to the control group, the culture medium containing ternary complexes exhibited lower absorbance values, indicating the inhibition of bacterial growth by the samples, as similarly reported by a previous study [10]. At equivalent culture durations, as the concentration of CA increased, absorbance values initially declined, followed by an elevation, with a turning point observed at 0.025% concentration. This suggested that the antimicrobial activity of the ternary complexes increased initially as CA concentrations fell below 0.025% and then decreased. The biphasic response in bacterial inhibition may be due to the fact that CA disrupts the bacterial TCA cycle, impacting both material and energy metabolism as well as cellular signal transduction, ultimately leading to metabolic disturbance and bacterial death. Given CA’s water-soluble nature, it struggles to permeate lipid bilayers directly and cannot directly rupture cell membranes [53]. Consequently, a substantial amount of CA enters cells via sodium ion channels to exert its effects [54]. The biological activity of PP primarily stems from its various bioactive peptides, and interactions with other food components such as polyphenols may influence the release and functionality of these peptides [55]. Research has shown that bioactive peptides within PP can bind to bacterial cell membranes, forming pores and disrupting cell integrity [56], thereby facilitating the entry of CA into cells and exhibiting antibacterial effects. Consequently, under appropriate CA concentration conditions, PP and CA can synergistically cooperate to exert antibacterial effects.

## 4. Conclusions

Stable non−covalent ternary complexes based on pea protein (PP, 0.5%), hyaluronic acid (HA, 0.125%), and chlorogenic acid (CA, 0~0.03%) at pH 7 were fabricated. The ternary complexes displayed nanometer−scale particles and high surface charge. CA showed good stability in the ternary complexes. All these complexes exhibited excellent emulsifying properties, antioxidative capacity, and antimicrobial activity, especially below 0.025% CA content. These findings provide valuable insights for the practical application of ternary composites in neutral foods and beverages.

## Figures and Tables

**Table 1:** The specific energy values (kcal/mol) and the molecules involved, along with their positions, in the formation of hydrogen bonds for different docking partners.

Docking Parents	ΔG	E_unbound_	E_VHD_	E_elec_	E_total_	E_torsional_	Hydrogren Bonds Formed
Legumin + HA	−4.85	−5.04	−4.35	−3.78	−5.04	3.28	Legumin:E:ASN325:HN: HA::UNK0:O
							Legumin:E:ARG323:HH12: HA::UNK0:O
							Legumin:E:LYS321:HZ1: HA::UNK0:O
							Legumin:E:ARG323:HH22: HA::UNK0:O
							Legumin:E:ARG115:HH11: HA::UNK0:O
Vicilin + HA	−3.56	−6.19	−5.26	−1.57	−6.19	3.28	Vicilin:C:LYS231:HN: HA::UNK0:O
							HA::UNK0:H: Vicilin:C:PRO205:O
							Vicilin:C:SER203:HN1: HA::UNK0:O
							Vicilin:C:GLY204:HN: HA::UNK0:O
Legumin + CA	−5.39	−4.62	−7.14	−1.54	−4.62	3.28	CA::UNL1:H: Legumin:F:VAL317:O
							Legumin:D:ASN438:HD21: CA::UNL1:O
							CA::UNL1:H: Legumin:F:ASN150:OD1
							Legumin:F:ASN150:HD21: CA::UNL1:O
Vicilin + CA	−5.55	−4.44	−7.48	−1.36	−4.44	3.28	Vicilin:C:HIS230:HD1: CA::UNL1:O
							Vicilin:C:LYS231:HN: CA::UNL1:O
							CA::UNL1:H: Vicilin:C:HIS230:O
							Vicilin:C:SER203:HN2: CA::UNL1:O

E_unbound_ represents the energy of the unbound system; E_VHD_ is the sum of van der Waals forces, hydrogen bonds, and energy from polar solvent interactions; E_elec_ denotes the energy of electrostatic interactions; E_total_ corresponds to the overall energy within the molecule; and E_torsional_ represents the torsional free energy.

## Data Availability

The original contributions presented in the study are included in the article, further inquiries can be directed to the corresponding author.
